# Nanopore long-read-only metagenomics enables complete and high-quality genome reconstruction from mock and complex metagenomes

**DOI:** 10.1186/s40168-022-01415-8

**Published:** 2022-12-02

**Authors:** Lei Liu, Yu Yang, Yu Deng, Tong Zhang

**Affiliations:** Environmental Microbiome Engineering and Biotechnology Laboratory, Center for Environmental Engineering Research, Department of Civil Engineering, The University of Hong Kong, Hong Kong SAR, China

**Keywords:** Nanopore sequencing, Long-read metagenomics, Activated sludge microbiome, Reference-quality genome reconstruction, NanoPhase, Prophage

## Abstract

**Background:**

The accurate and comprehensive analyses of genome-resolved metagenomics largely depend on the reconstruction of reference-quality (complete and high-quality) genomes from diverse microbiomes. Closing gaps in draft genomes have been approaching with the inclusion of Nanopore long reads; however, genome quality improvement requires extensive and time-consuming high-accuracy short-read polishing.

**Results:**

Here, we introduce NanoPhase, an open-source tool to reconstruct reference-quality genomes from complex metagenomes using only Nanopore long reads. Using Kit 9 and Q20+ chemistries, we first evaluated the feasibility of NanoPhase using a ZymoBIOMICS gut microbiome standard (including 21 strains), then sequenced the complex activated sludge microbiome and reconstructed 275 MAGs with median completeness of ~ 90%. As a result, NanoPhase improved the MAG contiguity (median MAG N50: 735 Kb, 44-86X compared to conventional short-read-based methods) while maintaining high accuracy, allowing for a full and accurate investigation of target microbiomes. Additionally, leveraging these high-contiguity reference-quality genomes, we identified 165 prophages within 111 MAGs, with 5 as active prophages, indicating the prophage was a neglected source of genetic diversity within microbial populations and influencer in shaping microbial composition in the activated sludge microbiome.

**Conclusions:**

Our results demonstrated that NanoPhase enables reference-quality genome reconstruction from complex metagenomes directly using only Nanopore long reads. Furthermore, besides the 16S rRNA genes and biosynthetic gene clusters, the generated high-accuracy and high-contiguity MAGs improved the host identification of critical mobile genetic elements, e.g., prophage, serving as a genomic blueprint to investigate the microbial potential and ecology in the activated sludge ecosystem.

Video Abstract

**Supplementary Information:**

The online version contains supplementary material available at 10.1186/s40168-022-01415-8.

## Introduction

The surge of shotgun sequencing data and advances in elaborating metagenomics approaches have drastically promoted our understanding of the diversity of microbial life [1]. In particular, genome-resolved metagenomics has been massively employed to unveil the black box of uncultured microbial majority, providing genome-level insights and expanding the tree of life [2] since the first metagenome-assembled genomes (MAGs) were reconstructed in 2004 [3]. However, even high-quality MAGs could be highly fragmented (worse in complex metagenomes) when assembled using the conventional short-read metagenomics, thus missing crucial genetic information [4]. To bridge the genome gaps, error-prone long reads generated on Nanopore and PacBio platforms were introduced, contributing to the leverage of hybrid (short- and long-read) sequencing strategy and the renaissance of high-contiguity reference-quality genome reconstruction from diverse microbiomes [5–9].

Quite different from Nanopore sequencing, PacBio sequencing could also generate high-accuracy (> 99%) HiFi reads with sacrificing read length and throughput, resulting in much higher sequencing costs in the metagenome project [10]. While the low entry and sequencing cost facilitates Nanopore sequencing accessible for most research labs, allowing a rapid turnaround time [11, 12]. Although Nanopore sequencing has difficulty fully characterizing long homopolymer regions, introducing insertion/deletion errors [13], the continuous improvement of sequencing accuracy, throughput and theoretically unlimited read length empower much more cost- and time-efficient genome reconstruction [14, 15]. To take advantage of the improvement of Nanopore platforms, here, we introduce NanoPhase, an open-source package to reconstruct reference-quality genomes efficiently from complex metagenomes and explore the metabolic potential based on the near-complete genomes.

## Methods

### Sample collection, DNA extraction, sequencing, and basecalling of Mock and activated sludge samples

The ZymoBIOMICS gut microbiome standard (catalog number: D6331) includes 21 microbial strains with varying abundances (0.0001–14%) to simulate the human gut microbiome. Microbial composition and reference genomes of the mock community can be accessed at https://www.zymoresearch.com/products/zymobiomics-gut-microbiome-standard. The activated sludge (AS) sample was collected on November 4th, 2019, from the aeration tank at the Shatin wastewater treatment plants in Hong Kong. The DNeasy PowerSoil Kit (Qiagen, Hilden, Germany) was used to extract DNA from mock and AS samples following the manufacturer’s protocols.

DNA sequencing of Mock and AS samples was on the GridION (Oxford Nanopore Technology, UK) platform using the Ligation Sequencing Kit (SQK-LSK109, the Kit 9 chemistry) on two and five flowcells (R9.4.1), respectively. In addition, another two flowcells (R9.4.1, one flowcell for the mock DNA and the other for the AS DNA) were used to generate long reads using the new Kit 12 (Q20+) chemistry. To evaluate the sequencing accuracy improvement of the Nanopore platforms, multiple Guppy versions with their best accuracy models were used for basecalling based on generated fast5 files, i.e., Guppy v3.0.3 (hac), Guppy v4.0.11 (hac), Guppy v5.0.16 (sup) and Guppy v6.0.0 (sup), releasing from April 2019 to December 2021. Guppy v5.0.16 (sup) showed the identical basecalling performance with Guppy v6.0.0 (sup), as the latter update was mainly for the Q20+ chemistry. Basecalled Nanopore raw reads were filtered based on the length of 1 Kb and the mean read identity of 80 (QA80), 90 (QA90) and 95 (QA95) using Filtlong (https://github.com/rrwick/Filtlong), respectively. The results present in the main text were all based on QA90 reads.

Besides the Nanopore sequencing, the same Mock and AS samples DNA were also delivered to Novogene Company Limited (Beijing, China) for short-read 150bp x 2 paired-end sequencing. In total, we obtained 53.7 Gb short reads for the Mock community and 53.6 Gb for the AS sample.

### Nanopore long-read-only framework to reconstruct genomes

**Fig. 1:**
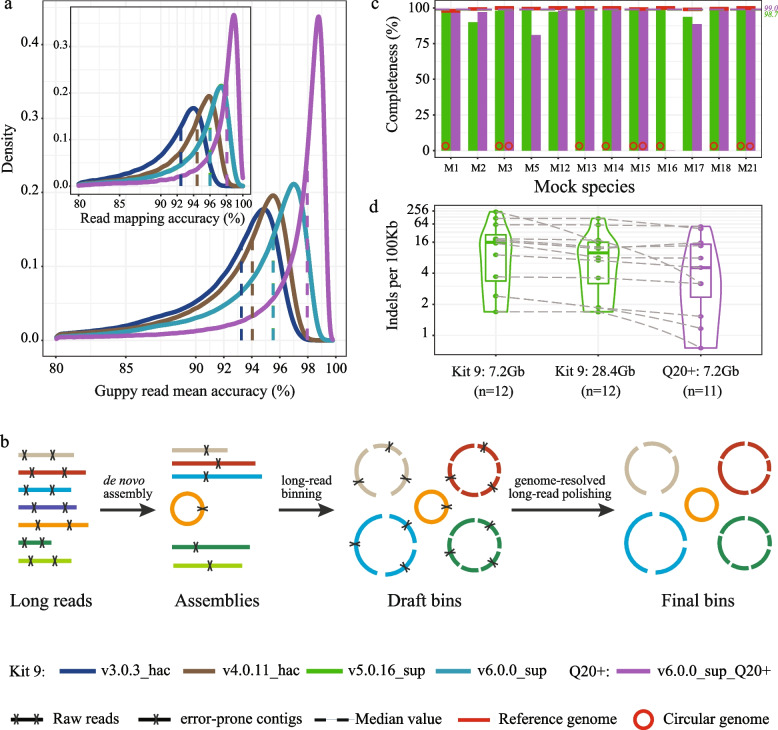
Performance evaluation of long-read-only framework based on a 21-strain human gut mock community. **a** Nanopore sequencing accuracy improvement due to the basecaller and chemistry upgrades. **b** NanoPhase framework to reconstruct reference-quality genomes. **c** Completeness and **d** Indels distribution of reconstructed genomes using datasets generated from the Kit 9 and Q20+ chemistry. The full name of Mock abbreviations in **c** can be found in Supplementary Table 1. The green and purple colors indicate genomes generated using Kit 9 and Q20+ chemistry, respectively

Figure 1b illustrates the brief framework of NanoPhase. In detail, metaFlye (v2.9-b1768) [16] was used to assemble filtered Nanopore long reads under the option firstly “--nano-hq -i 5 -g 4m” to generate assemblies. Then MetaBAT2 [17] and MaxBin2 [18] integrated with the coverage information were adopted to reconstruct two candidate genome sets, followed by the bin refinement step of MetaWRAP (v1.3.2) [19] to generate draft bins. Finally, long reads were mapped to the above draft bins using minimap2 (v2.21-r1071; map-ont) [20] with at least 90% identity and 90% coverage, producing draft-bin-based clusters. The long-read cluster was used to polish the draft bins individually with two rounds of Racon (v1.4.22) [21] and three rounds of medaka (v1.4.3; https://github.com/nanoporetech/medaka) to generate high-accuracy final bins. GNU Parallel [22] was used to speed up the analysis for parallel computation. This genome-resolved polishing would significantly improve the genome reconstruction efficiency, reducing polishing time and computational requirement, especially for deeply sequenced, complex metagenomes. However, we suggest including one round of Racon and one round of medaka polishing in the future, because additional rounds of long-read polishing were found to have deteriorated the genome quality slightly. Only MAGs with the completeness of above 50% and contamination of below 10% were retained for the downstream analysis.

### Short-read polishing and genome accuracy evaluation

The short-read dataset was also divided into different clusters by mapping short reads to polished final bins using minimap2 (-sr) with at least 90% identity and 90% coverage. Finally, the above final bins were polished using the short-read clusters using multiply rounds of Pilon [23]. The first round of Pilon polishing corrects the most errors, although additional rounds of Pilon polishing could further resolve the remaining errors but had little impact on the genome quality improvement.

Mapping read accuracy was estimated by mapping Nanopore reads to the mock reference genomes with blastn (v2.5.0) [24]. Misassembly rate and Indels of reconstructed MAGs were evaluated using QUAST (v5.0.2) [25] in the mock dataset. Completeness and contamination of reconstructed MAGs were appraised by CheckM (v1.0.80) [26]. True and observed homopolymer length distribution was computed by Counterr (https://github.com/dayzerodx/counterr). As no ground-truth genome standards for the AS metagenome, IDEEL [27], the fraction of predicted full-length proteins in each MAG, was used as an indirect indicator for evaluating genome accuracy with dependencies Prodigal (v2.6.3) [28] and Diamond (v2.0.13) [29]. Full-length proteins were defined when their length was more than 95% of the best-hit known protein [30] in the UniProt/TrEMBL [31] database (release 2021_04). Genome features, including rRNA operons and tRNA, were identified by Prokka (v1.14.6) [32]. Taxonomic assignment of MAGs was classified with GTDB-Tk (v1.6.0) [33] based on the GTDB R06-RS202 [34].

### Prophage and active prophage identification

Prophage sequences within reconstructed MAGs were determined and extracted using VIBRANT (v1.2.1) [35] with default parameters. Then, the prophage sequences with lengths more than 1Kb and their corresponding coordinates were used to estimate prophage activity using PropagAtE (v1.1.0) [36] by providing short reads.

## Results and discussion

The primary concern in the application of Nanopore sequencing is the error rate, and the median raw read accuracy for the R9.4 (the current most widely used version) was below 90% in 2019 [7]. However, based on the sequencing dataset of the mock community in this study, the median value of “Guppy read mean accuracy” has been substantially improved by the basecaller upgrades and newly developed chemistry, achieving 95.5% (Kit 9) and 98.0% (Q20+) basecalled by Guppy v6.0.0 (the sup model) (Fig. 1a). In addition, the density profile between “Guppy read mean accuracy” and “Read mapping accuracy” suggested that Nanopore read quality scores predicted by Guppy correlated well to the empirical read accuracy estimated from read-to-reference alignments, and some sequences quality was even underestimated (Fig. 1a and Supplementary Figure S1).

To facilitate rapid genome reconstruction, we proposed NanoPhase, a package to generate MAGs from a single long-read dataset (Fig. 1b). NanoPhase is designed to detangle the complex dataset into different clusters of draft bins and achieve genome-resolved efficient polishing. Totally, 28.4 Gb (N50: 5.9 Kb, two flowcells) and 7.2 Gb (N50: 5.4 Kb, one flowcell) were generated from the mock community using the Kit 9 and Q20+ chemistry, respectively. As expected, bacterial and archaeal genomes with sequencing coverage of < 5× cannot be reconstructed, and only one *E. coli* MAG was resolved to represent five closely related strains due to a very high average nucleotide identity (98.3–99.4%). Thus, 12 MAGs with median completeness of 98.7% were reconstructed from the Kit 9 dataset, and the Q20+ chemistry demonstrated slightly better performance, recovering 11 MAGs with median completeness of 99.0% (Fig. 1c). MAGs from both datasets were very close to reference genomes, benefiting from the read-accuracy and homopolymer resolution improvement (Supplementary Figure S2), which was also supported by low Indels errors (Fig. 1d) and high IDEEL [37] scores (Supplementary Figure S3). Notably, 8 (75%) MAGs were assembled into circular, complete genomes in the Kit 9 dataset, more than those generated from the Q20+ dataset (3), mainly due to a much higher sequencing coverage (~ 4-fold).

We next evaluated the genome reconstruction performance of NanoPhase to resolve a complex AS sample harboring thousands of microbial species. Both Kit 9 and Q20+ chemistries were used for noisy long-read sequencing on five and one flowcells, generating 85.3 (Kit 9 dataset, N50: 6.8 Kb) and 9.4 Gb (Q20+ dataset, N50: 6.5 Kb), respectively. In addition, we observed that filtration of sequencing reads with > 90% accuracy (QA90) is suitable for genome reconstruction in the complex microbiome, balancing yield and accuracy and generating more reference-quality MAGs (Supplementary Table 2).

**Fig. 2:**
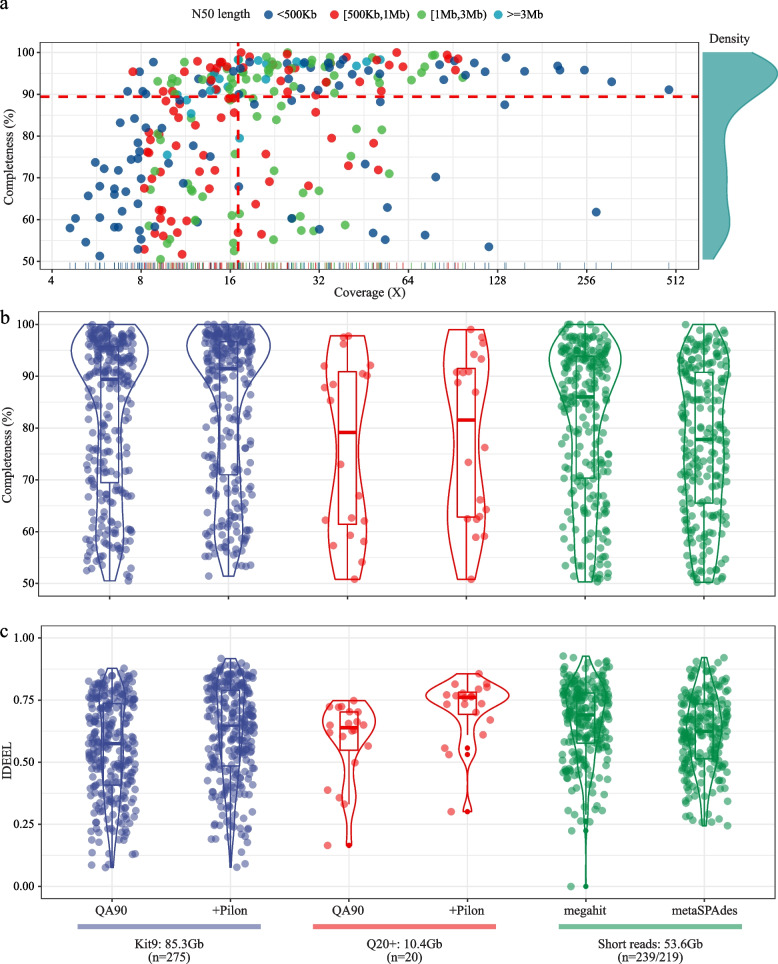
Reference-quality genome reconstruction from a complex activated sludge metagenome. **a** Genome quality distribution of reconstructed MAGs and their corresponding coverage using the Kit 9 dataset. **b** Distribution of the completeness and **c** IDEEL scores in different genome reconstruction strategies. “QA90” means read accuracy was above 90%. “+Pilon” means MAGs were polished with one round of Pilon correction. “megahit” and “metaSPAdes” indicate MAGs were reconstructed using short-read-based methods. The reconstructed genome numbers were presented in the bracket

Employing the Kit 9 dataset, 275 MAGs were reconstructed with the median completeness, contig count and coverage of 89.5%, 9 and 17X (Fig. 2a), representing 46.9% of the microbial community. Furthermore, the median N50 of these MAGs was 735 Kb, about 44- or 86-fold improvement compared to the short-read methods (Supplementary Table 3), demonstrating that genome gaps were remarkably closed by long reads. Of these MAGs, 94 MAGs with median coverage of 28X were classified as high-quality, fitting the stringent criteria of including full-length rRNA operons [38]. Notably, circular genomes were also recovered.

Compared to short-read-based methods, NanoPhase also reconstructed highly accurate genomes from the complex sample, from the aspect of completeness (median value of 89.5%; Fig. 2b) and IDEEL (median value of 0.58; Fig. 2c). As expected, the Q20+ chemistry performed better with higher IDEEL (median value of 0.64; Fig 2c) but only generated half of the flowcell (Kit 9 chemistry) throughput. Therefore, the yield limitation has increased the sequencing cost of the Q20+ chemistry in genome reconstruction at present. In addition, short-read-based Pilon polishing of the MAGs did not considerably improve the genome accuracy (Fig. 2b, c), suggesting that it is not an essential step in the future, particularly given the continuous improvement of Nanopore sequencing.

Besides the improved reconstruction of 16S rRNA genes and prediction of secondary metabolites potential discussed in our previous study [7], some mobile genetic elements identification that depended heavily on bacterial isolates could also benefit from these high-contiguity reference-quality genomes, e.g., prophage. As a major contributor to the diversity of bacterial gene repertoires, the relationship between bacteria and prophage is multifaceted, i.e., increasing bacterial fitness while at the risk of future lysis. In total, 165 prophages were identified within 111 MAGs with a median length of 14.3 Kb, and 1 MAG even possesses 5 different prophage sequences. The widespread prophages in recovered MAGs inferred that prophage was a critical factor in the evolution of microbial genomes via horizontal gene transfer between bacterial populations. Interestingly, no virulence factor and antibiotic resistance gene (ARG) were observed within recovered prophage sequences, suggesting that prophage mediated virulence factor and ARG transfer play a minor role in the studied activated sludge microbiome. In addition, the most prevalent accessory gene of prophage was methyltransferases, with a putative role in protecting prophages from the host immune system, which were found in 24 prophage sequences. Notably, most prophages remain dormant, transmitting vertically along with bacterial replication. However, 5 of them were determined as active prophages, indicating their host populations are undergoing prophage induction. Therefore, the active prophage lysis events may have altered the microbial community composition after sampling, suggesting the overlooked role of prophage in shaping the microbial community.

## Conclusions

Reference-quality genome reconstruction from complex metagenomes benefited significantly from long reads, promoting critical insights into “complete metagenomics” [39]. We efficiently reconstructed highly accurate and reference-quality genomes from mock and complex AS metagenomes by NanoPhase. Furthermore, the CheckM-based completeness of genomes generated from Nanopore long reads was even higher than those MAGs from conventional short reads, indicating that the error rates (mainly the Indels rate) in Nanopore sequencing reads were low enough to barely affect the genome quality at the protein (amino acid) level.

In addition, the superiority of NanoPhase was also supported by the high IDEEL score, which would further expand its promising application in genome-centric studies, providing a near-complete genomic blueprint and benefiting a finely detailed overview of diverse ecosystems. Currently, NanoPhase also supports genome reconstruction from bacterial isolates and antibiotic resistance genes (ARGs) identification from reconstructed MAGs based on the structured ARG database [40]. However, it cannot distinguish different microbial strains from metagenomics datasets, which would be the next step in the field.

Although sufficient coverage (~ 30×) is vital for the reference-quality genome reconstruction and challenging for most microbial populations in many natural complex communities, we expected that the extraction of ultra-high molecular weight (UHMW) DNA and methylation profiles might further reduce the requirement of sequencing depth and resolve strain-level genome reconstruction. Furthermore, integrating the Nanopore adaptive sampling strategy [41, 42], ultra-low-cost and accurate reference-quality genomes reconstruction to represent a near-complete microbial community is accessible from complex metagenomes. Ultimately, these reference-quality genomes are valuable harnesses for investigating microbial population potential, interaction, and evolution in the studied microbiomes.

## Supplementary Information

**Additional file 1: Table S1.** Mock ID and strain taxonomic information. **Table S2.** Assembly and genome reconstruction quality statistics generated from the Kit 9 dataset of the activated sludge microbiome by different cutoffs of read-accuracy filtering. **Table S3.** Genome reconstruction comparison between Nanopore long reads (Kit 9) and Illumina short reads. **Figure S1.** Guppy predicted mean read quality scores with different versions versus read mapping accuracy as measured by alignment to the reference genome. Nanopore raw reads were basecalled by a, Guppy v3.0.3 (hac), b, Guppy v4.0.11 (hac), c, Guppy v5.0.16/6.0.0 (sup) and d, Guppy v6.0.0 with the Q20+ chemistry (sup) then 10K reads were subsampled for the comparison. The orange dashed line indicates the perfect correlation between the two quantities. **Figure S2.** The homopolymer identification improvement of Nanopore raw reads due to the basecaller and chemistry upgrades. Nanopore raw reads were basecalled by a, Guppy v3.0.3 (hac), b, Guppy v4.0.11 (hac), c, Guppy v5.0.16/6.0.0 (sup) and d, Guppy v6.0.0 with the Q20+ chemistry (sup), then one million reads were subsampled for computing homopolymer identification by comparing the basecalled reads to the reference genome using Counterr. The black dashed line indicates the true homopolymer length. **Figure S3.** The IDEEL distribution of reconstructed MAGs from the Kit 9 and Q20+ datasets. The dashed line represents the IDEEL score of reference genomes. Genomes generated from the Q20+ chemistry showed better IDEEL scores.

## Data Availability

The Nanopore long-read ZymoBIOMICS gut microbiome standard dataset has been deposited in the NCBI database with BioProject accession number PRJNA804004. In addition, the activated sludge Nanopore long reads are available at NCBI with BioProject accession number PRJNA803959. The reconstructed 275 MAGs using only Nanopore long reads have been deposited in Figshare, allowing bulk download under DOI 10.6084/m9.figshare.20654796.
