# Supplementary material for: Nanopore long-read-only metagenomics enables complete and high-quality genome reconstruction from mock and complex metagenomes
Source: Microbiome. 2022 Dec 2;10:209. doi: 10.1186/s40168-022-01415-8 (PMC9716684; doi:10.1186/s40168-022-01415-8)
Supplement: Supplementary file 2 — Additional file 1: Table S1. Mock ID and strain taxonomic information. Table S2. Assembly and genome reconstruction quality statistics generated from the Kit 9 dataset of the activated sludge microbiome by different cutoffs of read-accuracy filtering. Table S3. Genome reconstruction comparison between Nanopore long reads (Kit 9) and Illumina short reads. Figure S1. Guppy predicted mean read quality scores with different versions versus read mapping accuracy as measured by alignment to the reference genome. Nanopore raw reads were basecalled by a, Guppy v3.0.3 (hac), b, Guppy v4.0.11 (hac), c, Guppy v5.0.16/6.0.0 (sup) and d, Guppy v6.0.0 with the Q20+ chemistry (sup) then 10K reads were subsampled for the comparison. The orange dashed line indicates the perfect correlation between the two quantities. Figure S2. The homopolymer identification improvement of Nanopore raw reads due to the basecaller and chemistry upgrades. Nanopore raw reads were basecalled by a, Guppy v3.0.3 (hac), b, Guppy v4.0.11 (hac), c, Guppy v5.0.16/6.0.0 (sup) and d, Guppy v6.0.0 with the Q20+ chemistry (sup), then one million reads were subsampled for computing homopolymer identification by comparing the basecalled reads to the reference genome using Counterr. The black dashed line indicates the true homopolymer length. Figure S3. The IDEEL distribution of reconstructed MAGs from the Kit 9 and Q20+ datasets. The dashed line represents the IDEEL score of reference genomes. Genomes generated from the Q20+ chemistry showed better IDEEL scores. [file 40168_2022_1415_MOESM1_ESM.docx]

**Supplementary Information for**

**Nanopore long-read-only metagenomics enables complete and high-quality genome reconstruction from mock and complex metagenomes**

Lei Liu, Yu Yang, Yu Deng, Tong Zhang*

Environmental Microbiome Engineering and Biotechnology Laboratory, The University of Hong Kong, Hong Kong SAR, China

Corresponding author: Tong Zhang; Phone: +852-28578551; E-mail: zhangt@hku.hk

**Table 1** | Mock ID and strain taxonomic information.

| Mock IDs | Mock species full name | Domain |
| --- | --- | --- |
| M1 | Akkermansia muciniphila | Bacteria |
| M2 | Bacteroides fragilis | Bacteria |
| M3 | Bifidobacterium adolescentis | Bacteria |
| M4 | Candida albicans | Fungi |
| M5 | Clostridioides difficile | Bacteria |
| M6 | Clostridium perfringens | Bacteria |
| M7 | Enterococcus faecalis | Bacteria |
| M8 | Escherichia coli (B-1109) | Bacteria |
| M9 | Escherichia coli (B-2207) | Bacteria |
| M10 | Escherichia coli (B-3008) | Bacteria |
| M11 | Escherichia coli (B-766) | Bacteria |
| M12 | Escherichia coli (JM109) | Bacteria |
| M13 | Faecalibacterium prausnitzii | Bacteria |
| M14 | Fusobacterium nucleatum | Bacteria |
| M15 | Lactobacillus fermentum | Bacteria |
| M16 | Methanobrevibacter smithii | Archaea |
| M17 | Prevotella corporis | Bacteria |
| M18 | Roseburia hominis | Bacteria |
| M19 | Saccharomyces cerevisiae | Fungi |
| M20 | Salmonella enterica | Bacteria |
| M21 | Veillonella rogosae | Bacteria |

**Table 2** | Assembly and genome reconstruction quality statistics generated from the Kit 9 dataset of the activated sludge microbiome by different cutoffs of read-accuracy filtering.

|  | **Nanopore long reads (Kit 9)** | | |
| --- | --- | --- | --- |
|  | QA80 | QA90 | QA95 |
| Data size (Gb) | 98.6 | 85.3 | 55.2 |
| N50 (Kb) | 6.8 | 6.8 | 6.9 |
| Median length (Kb) | 4.9 | 4.9 | 5.0 |
| Median accuracy (Q) | 13.3 | 13.8 | 14.8 |
| Assembly size (>1Kb, Gb) | 4.7 | 4.1 | 3.3 |
| No. of Circular contigs (length >0.5Mb) | 10 | 4 | 4 |
| No. of contigs (length >0.5Mb) | 959 | 958 | 704 |
| Contigs N50 (Kb) | 83.8 | 103.9 | 86.0 |
| Reads mapped to contigs (%) | 70.4 | 71.5 | 70.6 |
| No. of Closed-MAGs | 5 | 1 | 1 |
| No. of HQ-MAGs | 68 | 94 | 61 |
| No. of MQ-MAGs | 217 | 180 | 174 |
| Median contigs count/Closed-HQ MAGs | 9 | 9 | 7 |
| Median N50/Closed-HQ MAGs (Kb) | 963 | 924 | 1,011 |
| Closed-HQ MAGs size (Mb) | 314 | 418 | 263 |
| Mapped reads (%) of Closed-HQ MAGs | 14.5 | 19.9 | 14.4 |
| Median coverage of Closed-MAGs (X) | 17 | 36 | 25 |
| Median coverage of HQ-MAGs (X) | 29 | 28 | 23 |
| Total MAGs count | 290 | 275 | 236 |
| Mapped reads (%) of MAGs | 42.8 | 43.2 | 39.6 |
| Cost (US$) | 3000 | | |
| Cost per Closed-HQ MAGs (US$) | 41 | 32 | 48 |
| Cost per 90/5^a^ MAGs | 31 | 25 | 38 |

^a^MAGs with more than 90% completeness and less than 5%, which were assessed by CheckM.

**Table 3** | Genome reconstruction comparison between Nanopore long reads (Kit 9) and Illumina short reads.

|  | **Illumina** | | **Nanopore** |
| --- | --- | --- | --- |
|  | megahit | metaSPAdes | QA90 |
| Data size (Gb) | 53.6 | | 85.3 |
| Assembly size (>1Kb, Gb) | 2.3 | 2.3 | 4.1 |
| No. of Circular contigs (length >0.5Mb) | 0 | 0 | 4 |
| No. of contigs (length >0.5Mb) | 2 | 8 | 958 |
| Contigs N50 (Kb) | 4.5 | 3.4 | 103.9 |
| No. of Closed-MAGs | 0 | 0 | 1 |
| No. of HQ-MAGs | 7 | 8 | 94 |
| No. of MQ-MAGs | 232 | 211 | 180 |
| Total MAGs count | 239 | 219 | 275 |
| Median contigs count/MAG | 323 | 456 | 9 |
| Median N50/MAG (Kb) | 17 | 9 | 735 |


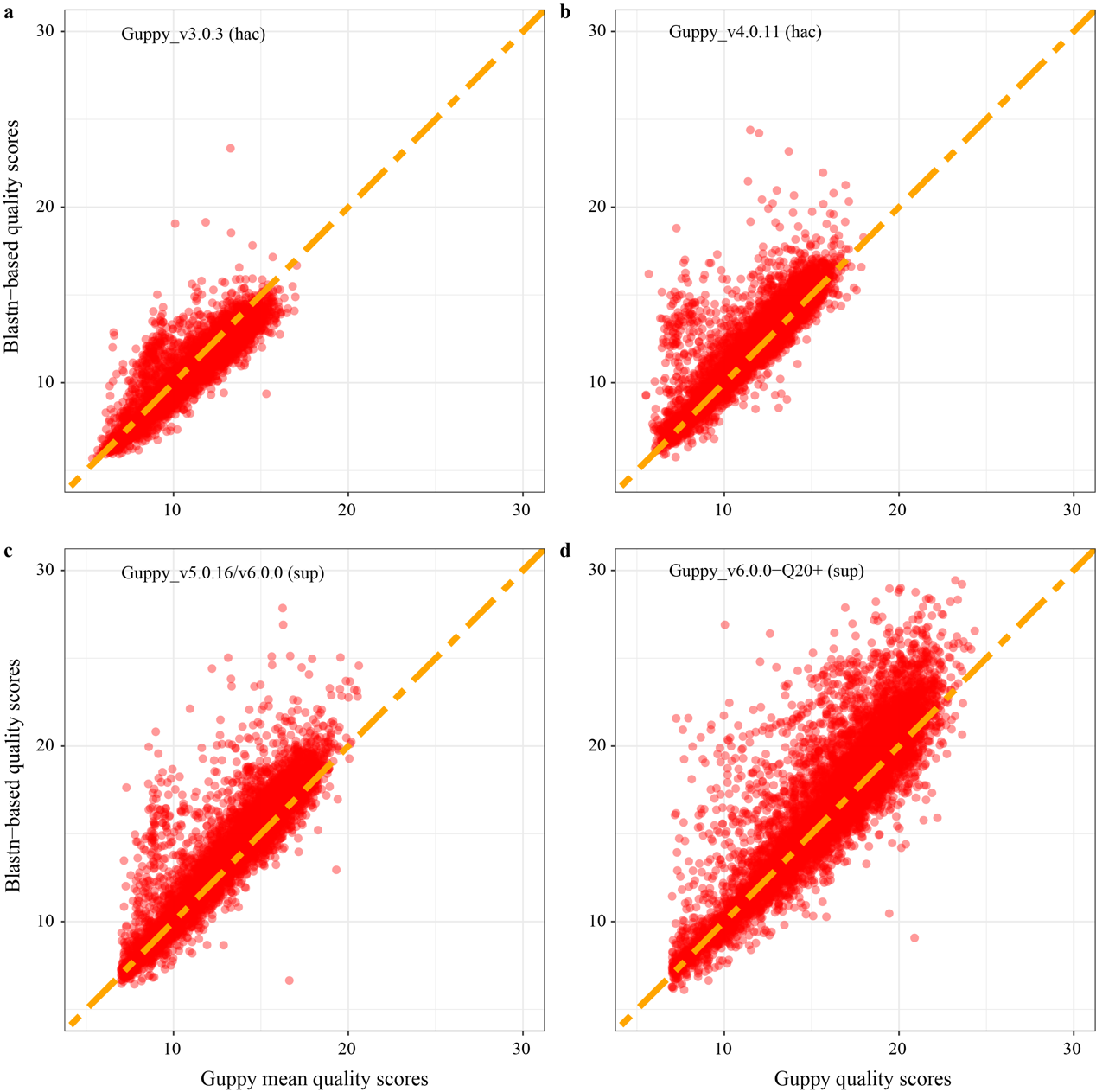


**Fig 1 | Guppy predicted mean read quality scores with different versions versus read mapping accuracy as measured by alignment to the reference genome.** Nanopore raw reads were basecalled by **a,** Guppy v3.0.3 (hac), **b,** Guppy v4.0.11 (hac), **c,** Guppy v5.0.16/6.0.0 (sup) and **d,** Guppy v6.0.0 with the Q20+ chemistry (sup) then 10K reads were subsampled for the comparison. The orange dashed line indicates the perfect correlation between the two quantities.


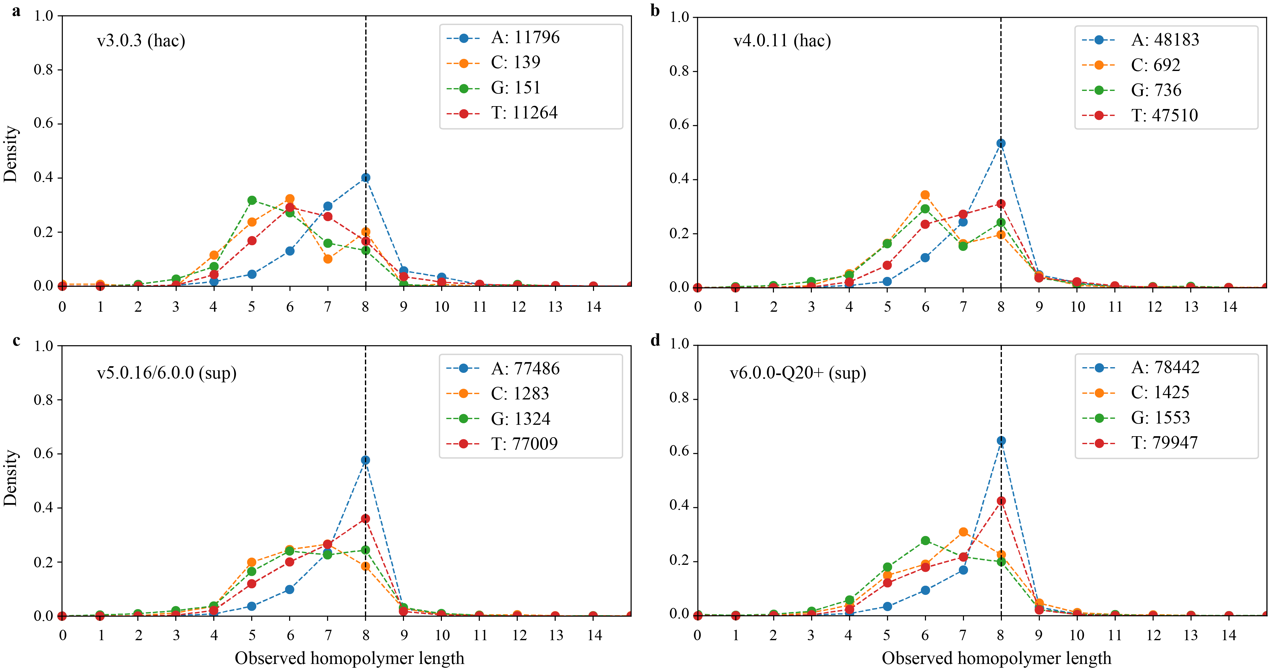


**Fig 2 | The homopolymer identification improvement of Nanopore raw reads due to the basecaller and chemistry upgrades.** Nanopore raw reads were basecalled by **a,** Guppy v3.0.3 (hac), **b,** Guppy v4.0.11 (hac), **c,** Guppy v5.0.16/6.0.0 (sup) and **d,** Guppy v6.0.0 with the Q20+ chemistry (sup), then one million reads were subsampled for computing homopolymer identification by comparing the basecalled reads to the reference genome using Counterr. The black dashed line indicates the true homopolymer length.


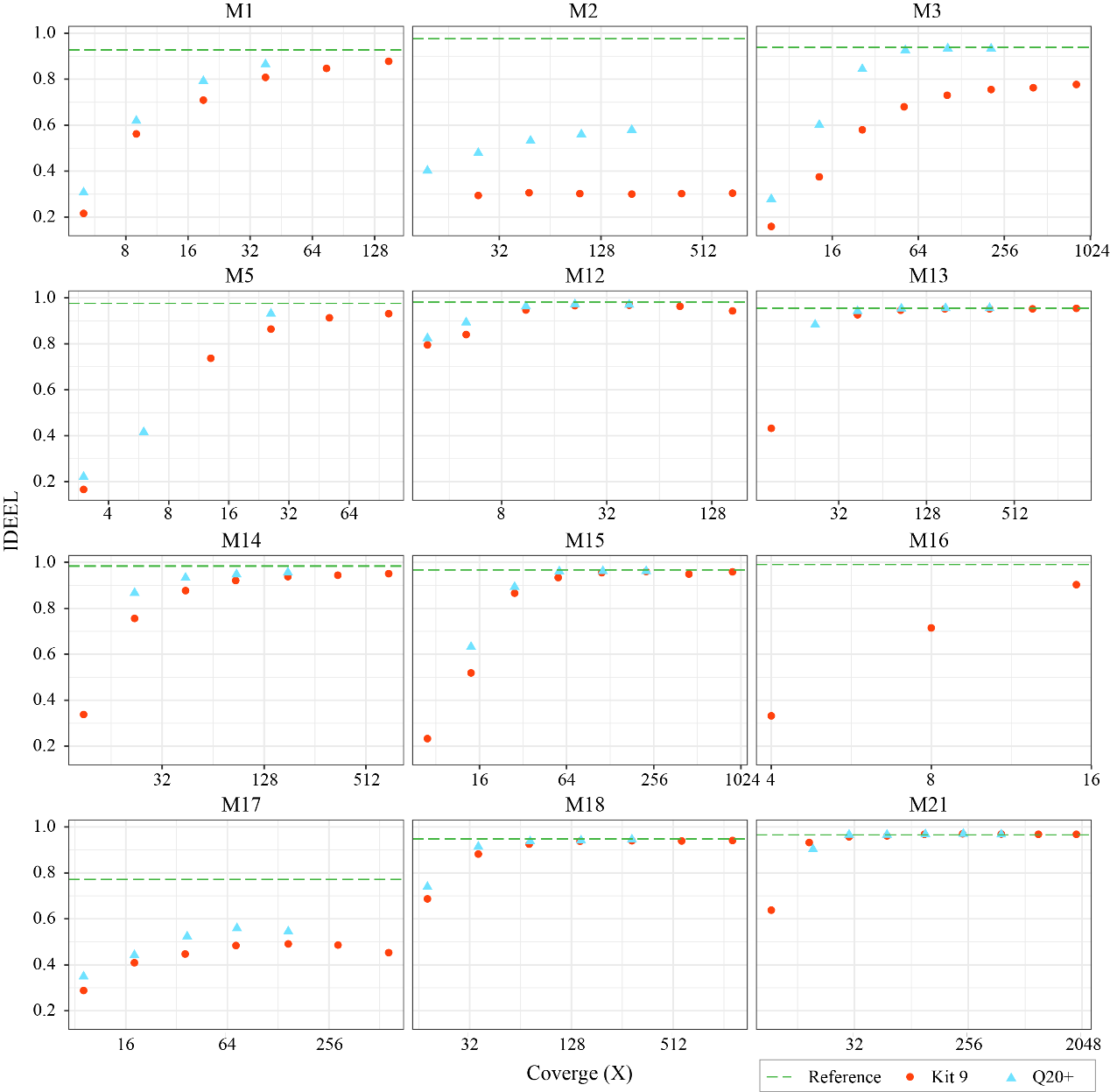


**Fig 3 | The IDEEL distribution of reconstructed MAGs from the Kit 9 and Q20+ datasets.** The dashed line represents the IDEEL score of reference genomes. Genomes generated from the Q20+ chemistry showed better IDEEL scores.
